# The Ideal Time for Iron Administration in Anemia Secondary to Blood Loss—An Experimental Animal Model

**DOI:** 10.3390/life11090898

**Published:** 2021-08-30

**Authors:** Mirela Tiglis, Ileana Peride, Lucian Cristian Petcu, Tiberiu Paul Neagu, Andrei Niculae, Alexandra Totan, Sabina Andrada Zurac, Ionel Alexandru Checherita, Ioana Marina Grintescu

**Affiliations:** 1 Department of Anesthesiology and Intensive Care, “Carol Davila” University of Medicine and Pharmacy, 020021 Bucharest, Romania; mirelatiglis@gmail.com (M.T.); ioana.grintescu@umfcd.ro (I.M.G.); 2 Department of Anesthesiology and Intensive Care, Emergency Clinical Hospital, 014461 Bucharest, Romania; 3 Department of Nephrology and Dialysis, “Carol Davila” University of Medicine and Pharmacy, 020021 Bucharest, Romania; ileana_peride@yahoo.com (I.P.); niculaeandrei@yahoo.com (A.N.); al.checherita@gmail.com (I.A.C.); 4 Department of Biophysics and Biostatistics, Faculty of Dentistry, “Ovidius” University, 900684 Constanta, Romania; petculucian@univ-ovidius.ro; 5 Department of Plastic Surgery and Reconstructive Microsurgery, “Carol Davila” University of Medicine and Pharmacy, 020021 Bucharest, Romania; 6 Department of Plastic Surgery and Reconstructive Microsurgery, Emergency Clinical Hospital of Bucharest, 014461 Bucharest, Romania; 7 Department of Biochemistry, Faculty of Dental Medicine, “Carol Davila” University of Medicine and Pharmacy, 020021 Bucharest, Romania; alexandra.totan@umfcd.ro; 8 Department of Pathology, “Colentina” Clinical Hospital, 020125 Bucharest, Romania; sabina.zurac@umfcd.ro; 9 Department of Pathology, Faculty of Dental Medicine, “Carol Davila” University of Medicine and Pharmacy, 020021 Bucharest, Romania

**Keywords:** experimental model, rats, acute bleeding, iron deficiency, intravenous iron, healing

## Abstract

Background: Anemia and iron deficiency are two of the main public health problems worldwide, associated with negative outcomes in surgical patients. This experimental study aimed to create a model of acute iron deficiency with anemia through blood loss and extensive surgery. Afterwards, intravenous iron was administered to correct the iron deficiency and to improve the hematological parameters in distinct moments regarding the surgical time. To assess the optimum time for therapeutic intervention, experimental subjects were compared, performing clinical, paraclinical, and histological examinations, as well. Methods: Male rats (*n* = 35), aged 11–13 months, were randomly designated into six groups. Anemia and iron deficiency were obtained through a 15% blood volume loss, followed by major surgical intervention (femur fracture and osteosynthesis using Kirschner wire). Therapeutic intervention was obtained with an intravenous ferric carboxymaltose infusion, as follows: group II: intraoperative (*n* = 7), group III: 48 h after surgery (*n* = 7), group IV: 48 h before surgery (*n* = 5), and group V: seven days before surgery (*n* = 6). Group I (*n* = 5) was left anemic, while group 0 (*n* = 5) was nonanemic without therapeutic intervention. Results and Discussion: In group I, serum iron lower than in group 0 (27.04 ± 6.92 μg/dL versus 60.5 ± 2.34 μg/dL), as well as hemoglobin (10.4 ± 0.54 g/dL versus 14.32 ± 2.01 g/dL) and ferritin values (22.52 ± 0.53 ng/mL versus 29.86 ± 3.97 ng/mL), validated the experimental model. Regarding wound healing after surgical trauma, we observed that neovascularization was more significant in group III, followed by group V, with fewer neutrophils, a well-represented and rich in lymphomonocytes inflammatory infiltrate associated with the biggest collagen fiber dimensions. The periosteal reaction and callus area presented thicker trabeculae in groups II and III compared to the anemic group. Conclusions: This original experimental study assessed the effect of perioperative intravenous iron administration at a specific time by comparing the weight, hematological, and iron status-defining parameters, as well as histological characteristics of the included subjects. The present findings highlight that correcting the iron deficiency in emergency settings through intravenous iron administration intraoperatively or 48 h postoperatively could determine the improved bioumoral parameters, as well as a better evolution of the postoperative wound and bone healing compared to the anemic group or subjects that received therapeutic intervention 48 h before surgery.

## 1. Introduction

Anemia and iron deficiency, despite being intensively explored, are two of the main public health problems worldwide, with certain negative impacts in hospitalized patients. Regarding anemia, its prevalence is about 26–75% in the preoperative period, reaching even the threshold of 90% postoperatively [1,2]. Iron deficiency is one of the leading causes of anemia in patients, a consequence of important blood losses, extensive surgeries, and poor gastrointestinal tolerance aggravated by the lack of time to perform a preoperative therapeutic intervention to optimize the hematological status [3,4].

For this reason, intravenous iron agents have been thoroughly researched and improved over the years [5] in order to be used as an alternative to a blood transfusion, a temporary measure, associated with various complications, in this subgroup of patients [6]. These new iron-containing agents have a safe profile, with stable molecules, which allows the administration of large amounts in a single infusion through a short period associated with fewer adverse events [5,7].

Various studies have shown the undisputed role of preoperative intravenous iron used for hemoglobin correction in patients undergoing elective surgeries [8,9,10]. Current practice involves correcting anemia ahead of selective major surgery, preferably for at least 30 days, knowing the negative causal relationship between anemia and postoperative outcome (increased morbidity and mortality, increased length of stay in the hospital, and increased rate of cardiovascular events) [1,10]. Regarding the immediate perioperative period when there is no time to properly correct anemia before surgery, the ideal moment for this intervention and its benefits on hemoglobin optimization is a field of continuous debate and research [11,12].

With reference to the healing process after a major surgical intervention, research has shown that anemia and iron deficiency impair skin healing through secondary hypoxia [13] and low initial inflammatory reaction [14], leading to inferior local tension and expansion resistance [15]. At the bone level, these clinical entities are associated with low mineral density and, therefore, a low resistance to fracture [16], decreased osteogenesis [17], and increased resorption [18].

From our knowledge, no experimental study has carried out a complex analysis of the response to iron supplementation depending on the time of therapeutic intervention in the immediate perioperative period. We performed in vivo studies to create an experimental model of acute anemia associated with an iron deficiency by blood volume losses in subjects undergoing major surgery. The primary aim of this study was to identify the best perioperative time to correct an iron deficiency in order to improve the hematological status and recovery in an emergency setting using intravenous iron pharmacological agents. Thus, the weight, bioumoral determinations, and morpho-pathological findings were compared in accordance with the outcome, wound, and bone healing. The secondary aim was to assess the influence of iron administration on kidney function, considering that iron-induced renal impairment was noticed in previous experimental studies [19].

## 2. Materials and Methods

### 2.1. Animal Care and Group Allocation

The animal care and experimental procedures strictly followed the regulations of the Sanitary Veterinary and Safety Food Direction of Bucharest (DSVSA), Romania, which authorized the Emergency Clinical Hospital of Bucharest Experimental Lab (No. 481/13.11.2020). The experimental study also received the approval of the Ethical Committee of the Emergency Clinical Hospital of Bucharest (No. 10404/23.11.2020). All experiments were performed according to the Animal Care and Use Committee guidelines and recommendations.

Healthy male adults Wistar rats, *rattus norvegicus* (INCD “Victor Babeș” Labs, Bucharest, Romania), with ages between 11 and 13 months, were left to acclimatize for 7 days. All animals were housed in polycarbonate cages (maximum 4 per cage), with sawdust bedding changed every three days under controlled environmental conditions (ambient humidity 40–60%, temperature 23 ± 2 °C, and dark/light cycle of 12 h), having access to standard food and water ad libitum.

After an adaptation period, thirty-five animals were randomly divided into six groups: group 0 (*n* = 5), the anemia model group (I) (*n* = 5), intraoperative iron group (II) (*n* = 7), 48-h postoperative iron group (III) (*n* = 7), 48-h preoperative iron group (IV) (*n* = 5), and 7 days preoperative iron group (V) (*n* = 6). These specific perioperative times were chosen to mimic emergency settings. Group 0 is the control group for the accuracy of weight and blood tests without intervention, while group I served as the control group for comparison with the other intervention groups.

### 2.2. Anemia Induction, Therapeutic Intervention, and Anesthetic and Surgical Procedures

**Figure 1:**
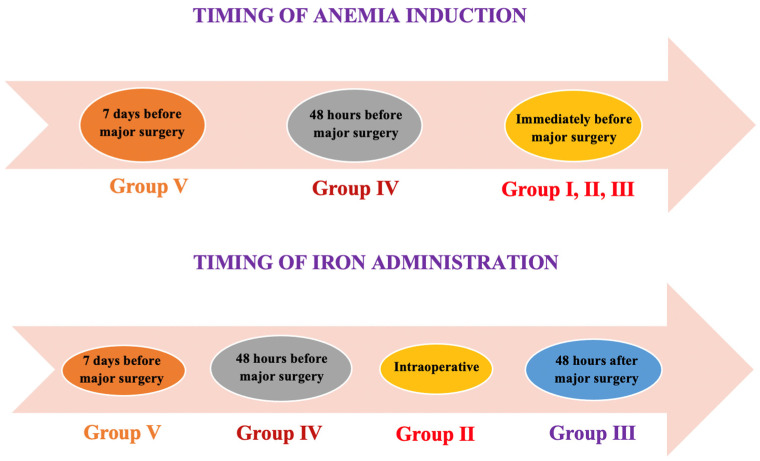
Timeline diagrams showing the time for anemia induction and iron administration in the studied groups. Legend: Group 0 is the control group without intervention for accuracy for weight and blood tests. Group I served as the control group for comparison with the other intervention groups.

The model of iron deficiency anemia was obtained through acute bleeding (dissection in the cervical area, internal jugular vein catheterization, and removal of approximately 15% of the total blood volume) of each subject followed by major surgery. In the treatment groups (II–V), animals were treated with intravenous ferric carboxymaltose (Ferinject^®^, Vifor Pharma, Paris, France) for iron deficiency, 20 mg/kg, through internal jugular vein catheterization as follows: group II: intraoperative, group III: 48 h after surgery, group IV: 48 h before surgery, and group V: seven days before surgery (Figure 1).

All surgeries were performed under general anesthesia, starting with isoflurane 1% (Rompharm Company, Otopeni, Romania) in an induction room until reflexes abolition, followed by the intraperitoneal administration of ketamine (Panpharma, Luitre, France)/xylazine (Eurovet Animal Health, Bladel, The Netherlands) 40/5 mg/kg. Surgical intervention had the subsequent steps: skin incision, dissection of the anatomical planes and exposure of the femur, intraoperative femur fracture, open reduction and osteosynthesis using Kirschner wire, anatomical planes closing, and wound dressing. All the animals were operated by the same surgical team, which was blinded to the group allocation. Rigorous hemostasis was obtained. Around 1 mL of blood loss was quantified using weighted gauzes. There were about four interventions/day with a duration of 50 min.

### 2.3. Clinical Evaluation

After surgery, every subject was single-housed in a cage and observed for 12 h. Weight was measured every two days after inducing anemia (day one for groups I–IV and day seven for group V) until day 7.

### 2.4. Blood Sampling and Biomarkers Determination

At the end of the experiment, the blood samples were collected. For iron profile determination, 2.4–2.6 mL of blood were collected in special tubes (PRIMA^®^ Vacuum Tube) for the serum separation (centrifugation for 10 min at 4 °C and stored at −20 °C until processing).

The level of ferritin and transferrin were measured using a semiautomatic biochemical analyzer (STAT FAX 303 PLUS^®^), as well as serum iron (A25 Biosystems^®^). The total iron-binding capacity (TIBC) and transferrin saturation (TSAT) were obtained through specific formulas (Table 1) [20].

Hemoglobin (Hb), hematocrit (Ht), red blood cells (RBC), platelets (PLT), mean cell volume (MCV), and mean cell hemoglobin (MCH) were directly measured using a veterinary hematology analyzer (Exigo H400 System^®^) after the tail puncture using manual micropipettes for minimal blood loss.

### 2.5. Histology

Promptly after blood sampling, at the end of the experiment (day seven: groups I–IV and day fourteen: group V), animals were sacrificed, and histological samples were harvested: the postoperative healed wound and surrounding skin, operated-on femur, and kidney. The samples were introduced in special recipients containing formol (Redmed Prodimpex, Bucharest, Romania) and left for fixation for 24 h. Further, the bone samples were decalcified using a nitric acid solution 15% for 12 h. Four micrometer sample slides were stained with hematoxylin and eosin (H&E) then evaluated using an electronic microscope (Olympus BX43^®^) equipped with a digital camera. The collected data were analyzed using Olympus cellSens Dimension^®^ software (version 1.13).

Three random images from each slide were photographed, and through a semi-quantitative histopathological analysis, the lymphomonocyte inflammatory infiltrate (extremely rare cells, rare, and relatively frequent) and collagen deposition (minimal, moderate, and marked) were graded. Collagen dimension fiber was measured in ten different zones from each sample and compared. Kidney probes were assessed to identify the presence/absence of iatrogenic lesions following intravenous iron administration. Pathologists were blinded to the group allocation.

### 2.6. Statistical Analysis

The results of the present study were analyzed with MedCalc (version 14) and IBM SPSS Statistics 23 programs. The procedures used were descriptive statistics (for characterizing discrete and continuous variables defined in the database), graphs, parametric statistical tests (one-way ANOVA test with the Student–Newman–Keuls test for all pairwise comparisons). The significance level used for the one-way ANOVA test is α = 0.05. The adjusted significance level used for multiple comparisons is α’ = 0.01. According to the test used, a *p*-value less than α (α’) should be considered of statistical significance.

## 3. Results

Three experimental subjects were excluded: one for wound dehiscence (group II), one for self-skin injuries (group III), and one for refracture of the operated femur (group V).

### 3.1. Clinical Findings

Regarding the animal body weights, there was no significant difference between anemic and nonanemic groups over the seven days after the intervention (*p* = 0.931) (Table 2).

### 3.2. Routine Blood Tests

At the end of the experiment (day seven or fourteen), the subject within group I presented the most modified red blood cell parameters (Table 3). According to ANOVA, at least two mean values were significantly different if the *p*-value < 0.05 and to determine which mean values were different, the Student–Newman–Keuls test was applied.

### 3.3. Iron Profile

The iron parameters that were investigated are presented in Table 4. Regarding the serum iron, the lowest value was registered in group I, followed by group IV. The lowest ferritin mean value was registered in group I, while the highest was registered in group III. The lowest value of transferrin was seen in group III, which was the closest to group 0. The lowest value of the TSAT was registered in group I, followed by group IV. The TIBC in group III was the closest to group 0.

### 3.4. Morphopathological Findings

#### 3.4.1. Wound Assessment

**Figure 2:**
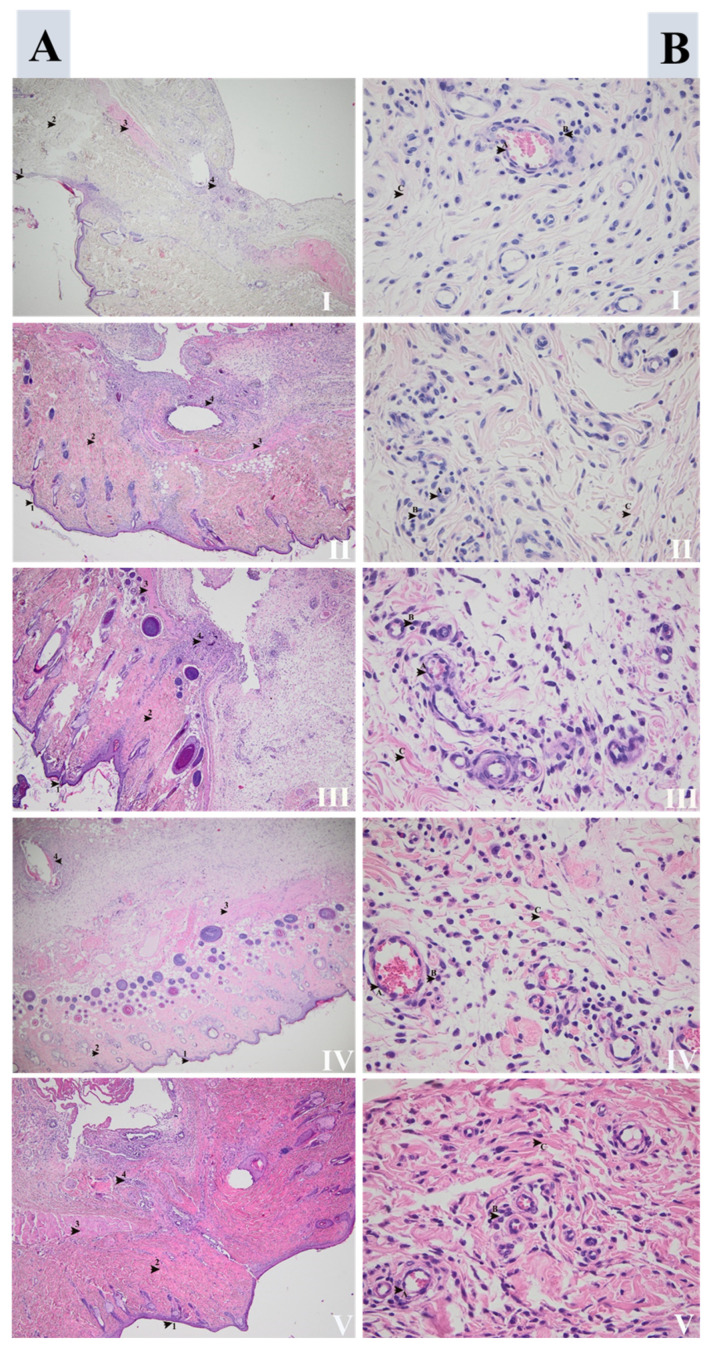
Skin repair after surgery in groups (I), (II), (III), (IV), and (V). (**Section** **A**) Overview: **1**: epidermis, **2**: dermis, **3**: skeletal muscle, and **4**: inflammatory infiltrate secondary to surgical incision (HE, 40×). (**Section** **B**) Detail images: (A) marks neovascularization more significant in groups (III), (V), and (II), followed by (IV) and (I). (B) Marks lymphomonocytes inflammatory infiltrates, better represented in groups (V), (III), and (II) compared with (IV) and (I). (C) Marks the collagen deposition that was moderate to marking in groups (V), (III), and (II), and minimal-to-moderate in groups (IV) and (I) (HE, 400×).

Skin and skeletal muscle fragments associated with granulation tissue, which disrupts the muscular layer, were found. In the granulation tissue, small areas of gigantocellular inflammation were observed surrounding some bone fragments or hair follicles, more prominent in groups II, III, and V (Figure 2–Section A). All the samples revealed neovascularization, fibroblastic hyperplasia, stromal collagen deposits, and lymphomonocyte inflammatory infiltrate with mixt cellularity. Collagen deposition was considered moderate to the marked in groups V, III, and II and minimal-to-moderate in groups IV and I. About the lymphomonocyte inflammatory infiltrate, it was relatively frequent in groups V, II, and III, rare in group IV, and extremely rare in group I (Figure 2–Section B). The mean values of the collagen fiber dimensions were the highest in group V (6.15 ± 2.34 μm), followed by group III (4.54 ± 0.95 μm) and group II (4.45 ± 0.92 μm), all within the normal limits for a newly closed wound. The lowest values were registered in group I (4.12 ± 0.92 μm) and group IV (3.99 ± 0.81 μm) (*p* = 0.004) (Table 5).

#### 3.4.2. Fracture Site Assessment

**Figure 3:**
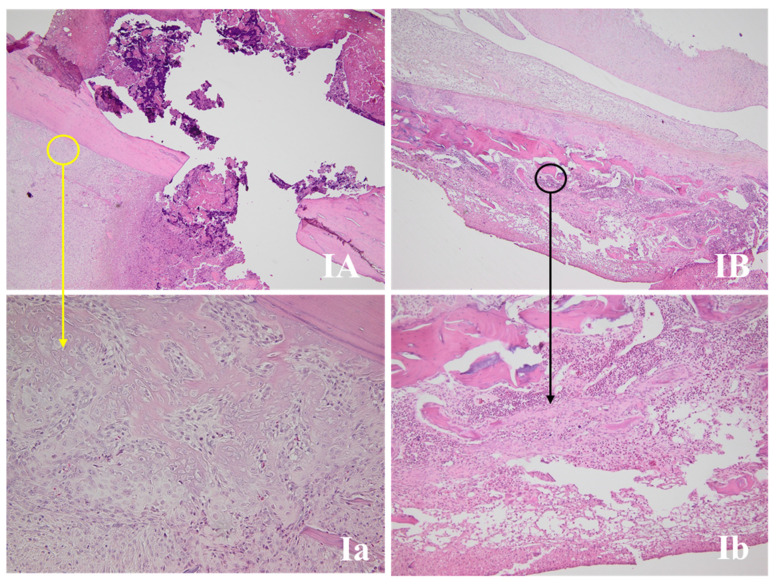
Bone repair of rat femurs in group I. (**IA**,**B**) present the fracture sites, with the yellow circle marking the periosteal reaction area and the black circle the bone repair reaction (callus) (HE, 40×). (**Ia**) Periosteal reaction with the thinnest trabeculae of all the samples (HE, 200×). (**Ib**) Thinner trabecular and a lesser degree of mineralization compared with the rest of the groups (HE, 100×).

**Figure 4:**
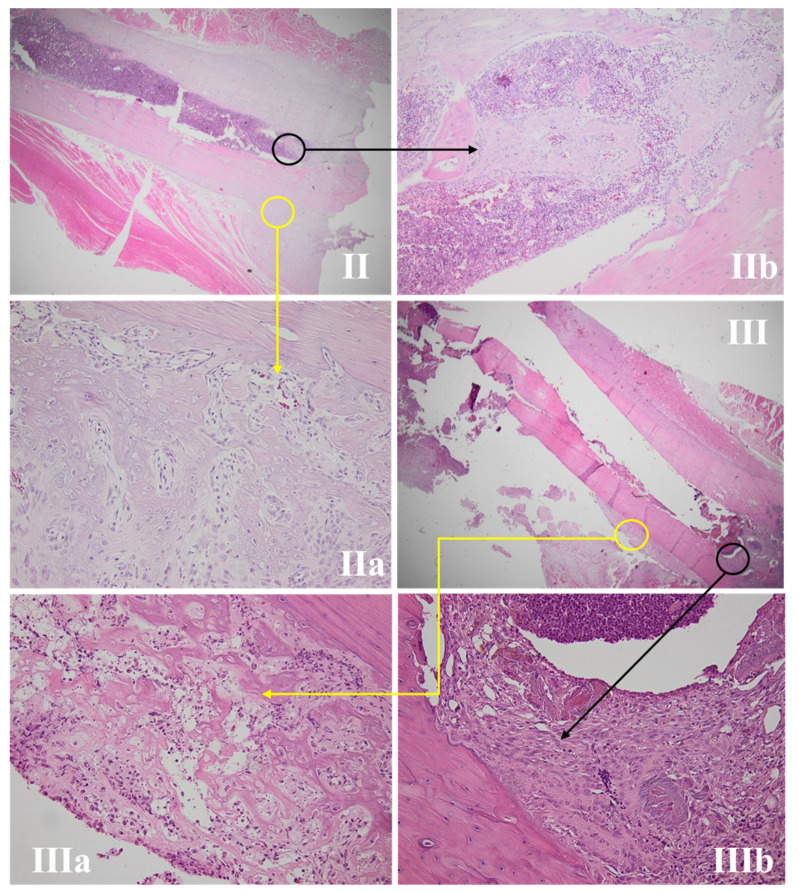
Bone repair of rat femur in groups II and III. (**II**,**III**) present the fracture sites, with the yellow circle marking a periosteal reaction area and the black circle marking the callus area (HE, 12.5×). (**IIa**,**IIIa**) expose the periosteal reaction, with the thickest trabeculae compared with groups IV and I and similar to group V (HE, 100×). (**IIb**,**IIIb**) show the bone repair reaction (callus), with thickest trabeculae and a better mineralization compared with groups IV and I and similar to group V (HE, 200×).

**Figure 5:**
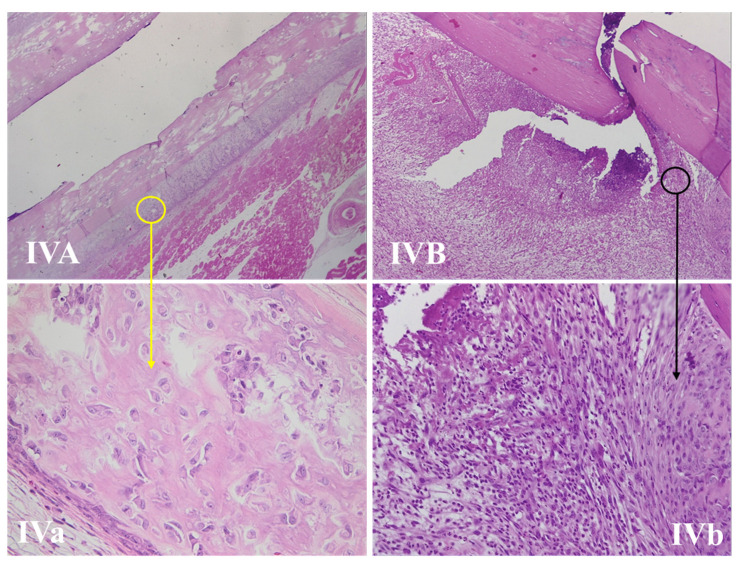
Bone repair of a rat femur in group IV. (**IVA,B**) The fracture sites, with the yellow circle marking the periosteal reaction area and the black circle marking the bone repair reaction (callus) (HE, 50×). (**IVa**) Periosteal reaction with the thinnest trabeculae compared with groups II, III, and V (HE, 400×). (**IVb**) Thinner trabecular and a lesser degree of mineralization compared with groups II, III, and V (HE, 400×).

**Figure 6:**
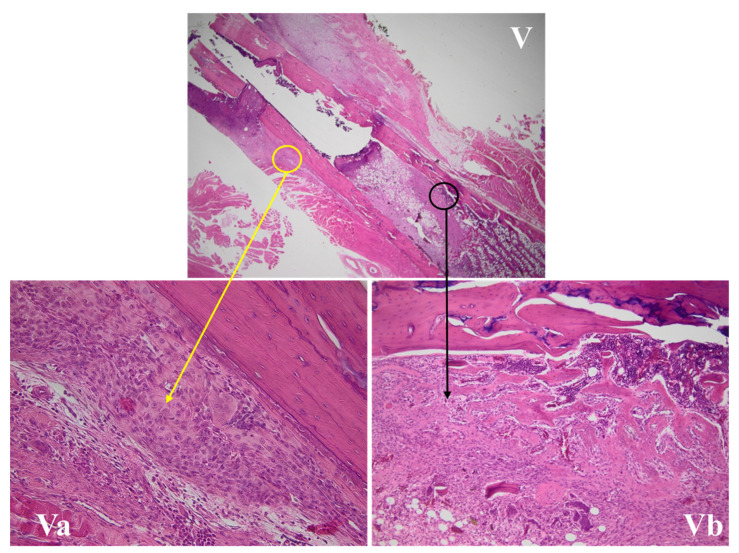
Bone repair of a rat femur in group V. (**V**) The fracture site, with the yellow circle marking a periosteal reaction area and the black circle the callus area (HE, 12.5×). (**Va**) The periosteal reaction, with the thickest trabeculae compared with groups IV and I and similar to groups II and III (HE, 200×). (**Vb**) The bone repair reaction (callus), with the thickest trabeculae and a better mineralization compared with groups IV and I, and similar to groups II and III (HE, 400×).

The fracture site, periosteal reaction, and callus area for all the studied groups are presented in Figure 3, Figure 4, Figure 5 and Figure 6. The periosteal reaction was observed, marked in groups II, III, and V (Figure 4 and Figure 6), with thicker trabeculae compared to group IV and I (Figure 3 and Figure 5). Taking into consideration the femur rat size, length of approximately 3.5 cm and thickness of 0.5 cm, the bone healing process presented some particularities. The induced femur bone trauma through fracture associated with Kirschner wire placement led to a bone repair reaction (callus) extended through the wire length, not only in the fracture site. The trabeculae were thicker in groups II, III, and V (Figure 4 and Figure 6) compared to groups IV and I, with a lesser degree of mineralization in the last (Figure 3 and Figure 5).

#### 3.4.3. Kidney Histological Assessment

**Figure 7:**
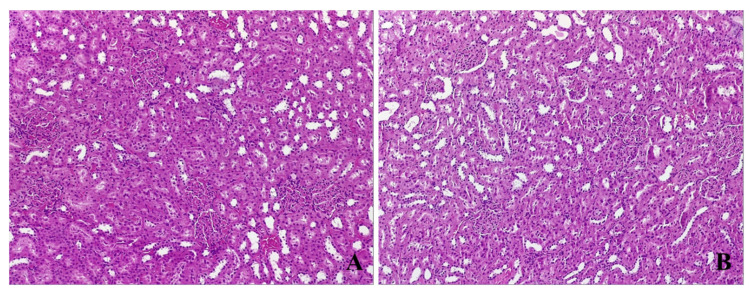
Renal samples: (**A**) anemic subject and (**B**) subject that received ferric carboxymaltose. The histological assessment shows no renal injury or iron deposition in these tissues regarding the iron administration or the time of intervention (HE, 100×).

No iron deposits or any renal injuries were identified in either of the groups (Figure 7).

## 4. Discussion

This study researched the proper time to administer intravenous iron in order to correct perioperative anemia and, thus, to improve the hemoglobin level in acute settings no longer than seven days before surgery or 48 h postoperative. To achieve that, we analyzed the weight, bioumoral markers, and histological skin and bone healing after a surgical trauma, and we observed that performing this intervention seven days before surgery, intraoperatively, or 48 h after surgery led to better results compared with 48 h before surgery or not performing it at all.

We obtained an iron deficit model associated with anemia by acute blood loss, a feasible experimental method according to the published data [21]. Specific indications were followed for group allocation [22], and all the possible variables were reduced to a minimum to respect the general recommendation regarding experimental animal studies [23,24]. This model was sustained by the fact that, in group I, the serum iron was lower than in group 0, the same as for the hemoglobin and ferritin values. In addition, the normal clinical examination and level of leucocytes, along with low ferritin in the anemic group and not higher than expected in the intervention group, were markers of infection absence [25].

Iron is an essential element involved in development and growth [26]. Animal studies have shown that iron deficiency leads to weight loss in anemic subjects after 9 days and becomes significant after around 17 days [21]. It is also a feature of chronic iron deficiency, especially through deprivation [27,28]. In our study, there were no differences regarding the animals’ weights, probably in relation with the acute nature of the events. Acute anemia and iron deficiency have unspecific symptoms like physical fatigue and decreased effort capacity [29,30], which may be thoroughly investigated in future research.

This study showed that seven days after an important blood loss, followed by major surgery, the hemoglobin, hematocrit, and red blood cells (sensitive and specific indices of anemia [31]) were significantly lower in group I compared with the groups where a therapeutic intervention was performed. The MCV and MCH values were close to group 0 in the subjects receiving intravenous iron, implying that, through a proper therapeutic intervention, the erythrocyte dimensions and hemoglobin content can be restored.

Another important finding was represented by higher platelets values in group I, significantly modified compared with the other groups. An analysis published by Hung et al. showed that the risk of adverse thromboembolic events grows from 7.8% to 15.8% in patients with thrombocytosis and iron deficiency [32]. Therefore, in patients undergoing major surgery, which itself induces a hypercoagulable state, the prompt correction of iron deficiency decreases this risk.

Clinical trials targeting postoperative wound healing are scarce and usually evaluate the local tissue strength [33,34]. In experimental studies, subjects with iron deficits induced by iron-free food products presented a lower tension and expansion resistance compared with nonanemic animals [35,36]. Immediately after injury (days one to seven), a primary local inflammatory state develops (abundant local neutrophils, monocytes, macrophages, and lymphocytes) [15], followed by neovascularization initiation [37]. From day four, the proliferative phase begins, with fibroblasts initiating collagen synthesis and deposition [38]. During the healing process, the amount of collagen and normal development of its fiber length and organization are responsible for the wound strength [37].

We observed that seven days after surgery, the neovascularization was more significant in group III, followed by groups V and II. Fewer neutrophils and a well-represented inflammatory infiltrate with lymphomonocytes were noticed in groups III, V, and II. The mean dimension values of the collagen fibers were the biggest in group V, followed by groups III and II. All of the above support the hypothesis that the wound healing process at seven days postoperative presents the best features in groups V, III, and II, while group I presents the lowest progress.

In bone tissue, iron is involved in the structure of some enzymes that synthesize collagen [39] and in vitamin D metabolism [40]. There are few clinical or experimental studies analyzing the interconnection between the iron status and bone healing, especially rapidly after an injury or a surgical procedure. Some studies have shown that low levels of ferritin are associated with low bone mineral density [41] or that an iron deficiency induces bone resorption acceleration [18]. After an injury, blood vessels and normal bone architecture disruption start an inflammatory cascade, with local hematoma formation [42]. Consequently, devitalized bone resorption starts, followed by neovascularization, new bone formation on either side of the fracture site with a callus appearance [43], cartilaginous tissue, mineralization, and bone remodeling [44]. In this study, the periosteal reaction presented thicker trabeculae in groups II, III, and V, compared with groups IV and I. The same aspects were observed at the callus area, with a higher degree of mineralization being noticed in groups II, III, and V in comparison with the anemic group (I) and group IV. Therefore, it can be stated that correcting iron deficiency through an intraoperative or 48-h postoperative intervention presents a more favorable bone-healing trend in comparison with the other studied groups.

Studies have shown that many iron-containing intravenous agents have metabolites with renal excretion, therefore having the potential of producing nephrotoxicity through oxidative stress [45]. In order to test our secondary hypothesis, a renal histopathological assessment was performed, and no kidney injury was observed in group I, nor in any subject receiving intravenous ferric carboxymaltose.

The study limitations counted the small number of subjects per group that could impair the statistical power and the short duration of the experiment. Additionally, using specific markers for inflammation and infections will increase the value of the research. Further investigation of these aspects above the acute perioperative period (7 days after surgery) will help compare the long-term benefits of these interventions.

## 5. Conclusions

This study presents an original experimental model of acute induced anemia and iron deficiency through blood loss followed by major surgery. From our knowledge, it is the first experimental study to assess the effect of perioperative intravenous iron administration in an emergency setting at a specific time by comparing the weight, hematological, and iron status-defining parameters, as well as the histological characteristics of the included subjects seven days after surgery. Rapidly correcting the iron deficiency leads to a favorable improvement of hemoglobin, iron homeostasis and clinical outcome in surgical subjects. The present findings show that, by correcting the iron deficiency through intravenous iron administration 7 days before surgery, intraoperative, or 48 h postoperatively, a better clinical outcome is achieved, including a better healing process evolution of postoperative wounds and bone fractures compared to the anemic group and subjects that received therapeutic intervention 48 h before surgery.

## Figures and Tables

**Table 1:** Calculation formulas for the TSAT and TIBC.

Parameter	Formula	Standard Measurement Unit
TSAT	= 100 × serum iron/serum transferrin × 1.25	%
TIBC	= serum transferrin (mg/dL) × 1.25	μg/dL

TSAT = transferrin saturation and TIBC = total iron binding capacity.

**Table 2:** Experimental subject mean weight variations during the study period.

	Weight	Day 1 (G-I–GIV) (Day 7 for G-V) after Anemia and Surgery, Reference Values for G-0		Weight Day 3 after Surgery		Weight Day 5 after Surgery		Weight Day 7 after Surgery	
Group	N	Mean	SD	Mean	SD	Mean	SD	Mean	SD
G-I	5	363	28	354	30	346	24	349	29
G-II	6	350	29	340	31	336	33	343	28
G-III	6	349	43	340	46	338	47	341	44
G-IV	5	355	34	348	33	347	33	349	36
G-V	5	364	33	358	32	357	31	359	32
G-0	5	340	44	-	-	-	-	-	-
	F-ratio * (*p*)	0.334 (0.888)		0.276 (0.890)		0.306 (0.871)		0.208 (0.931)	

* One-way ANOVA test. G-0: group 0, G-1: group I, G-II: group II, G-III: group III, G-IV: group IV, and G-V: group V.

**Table 3:** Effects of therapeutic intervention on routine blood test parameters (the results values are conferred as mean ± SD).

Group	Hb (g/dL)	Ht (%)	PLT (10^9^/L)	RBC (10^12^/L)	MCH (pg)	MCV (fL)
G-0 (*n* = 5)	14.32 ± 2.01	36.88 ± 4.81	227.20 ± 27.87	7.64 ± 0.94	18.70 ± 0.39	48.28 ± 0.98
G-I (*n* = 5)	10.4 ± 0.54	23.62 ± 1.23	518.80 ± 56.53	5.56 ± 0.11	18.44 ± 0.54	41.98 ± 1.51
G-II (*n* = 6)	13.15 ± 0.61	34.58 ± 1.15	300 ± 23.41	7.01 ± 0.32	18.78 ± 0.50	49.25 ± 2.38
G-III (*n* = 6)	12.2 ± 0.33	32.67 ± 1.63	277.33 ± 43.54	6.35 ± 0.23	19.32 ± 0.67	51.75 ± 2.87
G-IV (*n* = 5)	12.72 ± 0.54	34.18 ± 1.50	276.60 ± 33.66	6.74 ± 0.38	18.94 ± 0.77	50.82 ± 2.48
G-V (*n* = 5)	14.26 ± 1.51	37.34 ± 3.43	316.20 ± 17.37	7.56 ± 0.78	18.88 ± 0.86	49.56 ± 3.40
F-ratio * (*p*)	9.368 (<0.001)	18.768 (<0.001)	40.447 (<0.001)	11.031 (<0.001)	1.146 (0.361)	10.532 (<0.001)
Group 0 different from **	I, III	I	I, II, III, IV, V	I, III, IV	-	I

G-0: group 0, G-1: group I, G-II: group II, G-III: group III, G-IV: group IV, G-V: group V, Hb: hemoglobin, Ht: hematocrit, PLT: platelets, RBC: red blood cells, MCH: mean cell hemoglobin, and MCV: mean cell volume. * One-way ANOVA test. ** Student–Newman–Keuls test for all pairwise comparisons (*p* < 0.01).

**Table 4:** The effect of therapeutic intervention on the serum iron, ferritin, transferrin, TSAT, and TIBC (the results values are conferred as the mean ± SD).

Group	Serum Iron (μg/dL)	Ferritin (ng/mL)	Transferrin (ng/mL)	TSAT (%)	TIBC (μg/dL)
G-0 (*n* = 5)	60.50 ± 2.34	29.86 ± 3.97	67.54 ± 7.14	72.42 ± 9.06	84.38 ± 8.95
G-I (*n* = 5)	27.04 ± 6.92	22.52 ± 0.53	88.28 ± 7.65	23.30 ± 6.53	110.36 ± 9.59
G-II (*n* = 6)	61.50 ± 11.43	28.73 ± 3.03	71.85 ± 9.74	68.93 ± 12.47	89.80 ± 12.18
G-III (*n* = 6)	55.95 ± 7.26	38.00 ± 4.89	71.05 ± 6.65	63.05 ± 5.99	88.77 ± 8.30
G-IV (*n* = 5)	38.84 ± 12.34	27.34 ± 4.01	77.26 ± 8.24	40.20 ± 13.01	96.52 ± 10.35
G-V (*n* = 5)	71.68 ± 21.68	28.38 ± 1.59	105.24 ± 12.51	56.62 ± 13.89	127.40 ± 23.41
F-ratio * (*p*)	9.887 (<0.001)	12.143 (<0.001)	13.353 (<0.001)	16.475 (<0.001)	8.182 (<0.001)
Group 0 different from **	I, IV, V	I, III	I, V	I, IV, V	I, V

TSAT: transferrin saturation, TIBC: total iron-binding capacity, G-0: group 0, G-1: group I, G-II: group II, G-III: group III, G-IV: group IV, and G-V: group V. * One-way ANOVA test. ** Student–Newman–Keuls test for all pairwise comparisons (*p* < 0.05).

**Table 5:** Collagen fiber dimension (µm) mean values for the study groups (I–V) (the result values are conferred as the mean ± SD).

Group	Collagen Fiber Dimension (µm)	Different from Group nr. **
G-I	4.12 ± 0.92	V
G-II	4.45 ± 0.69	V
G-III	4.54 ± 0.95	V
G-IV	3.99 ± 0.81	V
G-V	6.15 ± 2.34	I, II, III, IV
F-ratio * (*p*)	4.549 (0.004)	

G-1: group I, G-II: group II, G-III: group III, G-IV: group IV, and G-V: group V. * One-way ANOVA test. ** Student–Newman–Keuls test for all pairwise comparisons (*p* < 0.01).
